# The East African highland cooking bananas ‘Matooke’ preferences of farmers and traders: Implications for variety development

**DOI:** 10.1111/ijfs.14813

**Published:** 2020-10-08

**Authors:** Kenneth Akankwasa, Pricilla Marimo, Robooni Tumuhimbise, Moreen Asasira, Elizabeth Khakasa, Innocent Mpirirwe, Uli Kleih, Lora Forsythe, Geneviève Fliedel, Dominique Dufour, Kephas Nowakunda

**Affiliations:** ^1^ National Agricultural Research Laboratories (NARL) P.O. Box 7065 Kampala Uganda; ^2^ Alliance of Bioversity International and International Centre for Tropical Agriculture (CIAT) Kampala 24384 Uganda; ^3^ Rwebitaba Zonal Agricultural Research and Development Institute Fort Portal 96 Uganda; ^4^ Natural Resources Institute (NRI) University of Greenwich Central Avenue, Chatham Maritime Kent ME4 4TB UK; ^5^ International de Recherche Agronomique pour le Development (CIRAD) UMR Qualisud Montpellier F‐34398 France; ^6^ Qualisud Univ Montpellier Montpellier SupAgro Univ d'Avignon Univ de La Réunion CIRAD, UMR QualiSud Montpellier F.34398 France

**Keywords:** End‐user, gender, highland bananas, *Matooke*, quality characteristics, Uganda, variety development

## Abstract

‘Matooke’ is a staple food made from Highland cooking bananas in the Great Lakes region of East Africa. Genetic improvement of these bananas for resistance to pests and diseases has been a priority breeding objective. However, there is insufficient information on fruit quality characteristics that different users prefer, resulting in sub‐optimal adoption of new varieties. This study identified matooke characteristics preferred by farmers and traders, using survey data from 123 farmers, 14 focus group discussions and 40 traders. Gender differences were considered. The main characteristics that were found to drive variety preferences were agronomic (big bunch, big fruits) and quality (soft texture, good taste, good aroma, yellow food). There were minimal geographical and gender differences for trait preferences. Quality characteristics need to be defined in terms of physical–chemical underpinnings so that breeding programmes can apply accurate high‐throughput systems, thereby improving adoption and impact of new banana varieties.

## Graphical abstract

Limited information of fruit quality characteristics preferred by different users of East African Highland Cooking Bananas constrains their genetic improvement. This study identified big bunches and fingers, soft texture, good taste, aroma and yellow colour of the cooked products as key characteristics for farmers and traders

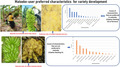

## Introduction

The East African Highland Cooking Bananas (EAHCBs), also known as*’*Matooke^1^’ bananas, support livelihoods of close to 30 million people, mainly smallholder rural farmers in the Great Lakes region of East Africa (Nyombi, 2013; NBRP, 2018). With production estimated at 10 metric tonnes (MT) per year (FAO, 2001; Lusty and Smale, 2002), the crop is mainly grown by smallholder producers on an average of 0.3 hectares (Bagamba, 2007). About 70% of matooke production is consumed at household level while 30% is sold through agents/brokers, wholesalers and retailers to the urban consumers as bunches, clusters or fingers (Akankwasa *et al*., 2013).

Banana production and consumption are deeply embedded in the Ugandan culture, where some varieties have cultural roles among the farming communities (Karamura, 1998; NBRP, 2016). In most cases, men manage the banana plantations for cash while women manage those used directly for household food. However, even in the same plantation, individual bunches may be claimed by men for sale or by women for direct food use (NBRP, 2016; Nalunga *et al*., 2015).

Matooke bunches are harvested at fruit mature green stage, peeled, wrapped in banana leaves, steamed or boiled, mashed, then typically eaten with or without a sauce. The fruits may also be eaten directly after boiling or steaming (FAO, 2018; Marimo *et al*., 2019; Nowakunda *et al*., 2019). This is the most common food in Uganda and other areas in the Great Lakes region. When cooked, Matooke is characterised by a unique flat taste and aroma, golden yellow colour and a soft texture. These characteristics constitute the unique quality described as ‘tookeness’ (NBRP, 2016), originating from the term ‘Matooke’. Consumers desire these attributes in new varieties (Akankwasa *et al*., 2013).

Cultivation of the EAHCBs is increasingly becoming challenging due to a host of pests and diseases that reduce yield and quality (Kalyebara *et al*., 2007; Tushemereirwe *et al*., 2003; Gold *et al*., 1999) and other environmental factors. This has necessitated investments into breeding programmes. Currently, matooke breeding is done through conventional means by crossing fertile female landraces with wild male parents (Nyine *et al*., 2017; Tumuhimbise *et al*., 2018). This process often introduces undesirable characteristics into hybrids (Khan *et al*., 2009). Without multiple generations of crossing and selection, breeders rarely generate hybrids with good user acceptance. Even then, over 90% of generated hybrids are often rejected by users (Morris & Bellon, 2004; Tumuhimbise *et al*., 2016).

To raise the chances of obtaining a hybrid with acceptable qualities, breeders typically generate thousands of clones and evaluate them with farmers (Tumuhimbise *et al*., 2016). The process of selection and getting feedback from users is lengthy and costly, lasting over 10 years. Most of the hybrids generated are often rejected mainly because they do not meet end‐users needs (Bechoff *et al*., 2018; Tumuhimbise et al., 2019). Breeders focus on generating hybrids that have improved resistance and agronomic characteristics, with less consideration the user’s preferences (Bechoff *et al*., 2018) earlier on in the breeding cycle. End‐user preferences are often captured at the end of the breeding cycle when varieties have already been developed and feedback might be too late. Evaluation at this stage involves on‐farm trials and the use of sensory methods to determine the acceptance (Ssemwanga, 1996; Nowakunda *et al*., 2000; Nowakunda & Tushemereirwe, 2004; Akankwasa *et al*., 2013; Tumuhimbise *et al*., 2018).

Studies on hybrid evaluation and adoption in cassava, banana and rice hybrids show that despite the huge investments, adoption rates for hybrids are low as often they do not meet market needs that are driven by end‐user preference (Sebasigari, 1996; Bechoff *et al*., 2018; Joshi & Bauer, 2006; Smale, & Tushemereirwe, 2007; Asante, 2013; Thiele *et al*., 2021). Farmers’ perceptions and experiences about the attributes of varieties are important factors that influence their variety use decisions (Wale, 2012). Edmeades (2003); Hintze *et al*. (2003); and Wale (2012), reported that varieties that lack farmer demanded characteristics were not retained on farmers’ fields. Marimo *et al*., 2020 provide a comprehensive review of studies that document banana trait preferences of various value chain actors in Sub Saharan Africa. Documentation on specific preferred quality characteristics of matooke is lacking thus making it difficult for breeders to have appropriate guidance during hybrid development and selection. Breeding programmes should engage end‐users to learn about the traits they prefer for incorporation into the breeding process.

The aim of this study is to identify preferred and less preferred characteristics of *Matooke* by farmers and traders to guide breeders towards appropriate selection criteria for varieties that would have high adoption rates.

## Materials and methods

The study was conducted in two districts of Uganda (Nakaseke and Mbarara) where matooke bananas are a staple crop (NBRP, 2016). Nakaseke district which is in the central region is traditionally described as a coffee–banana farming system. It falls within the Central Wooded Savannah agro‐ecological zone with an altitudinal range of 1086–1280 masl, mean annual rainfall of up to 1100 mm and temperatures ranging from 16 ^◦^C to 30 ^◦^C (Mulumba *et al*., 2012). It is also described as a low production area (<7.0 metric tonnes per hectare per year) with high intensity of defoliating diseases such as black Sigatoka and pests like weevils and nematodes (Tushemereirwe *et al*., 2003). However, this area is closer to large end markets for cooking bananas in Uganda. Nakaseke is in a region which is a primary target for promotion of newly bred resistant hybrids. Mbarara district in western region on the other hand, is described as a predominantly banana–cattle farming system (Mulumba *et al*., 2012). It is a high banana production area (>18 metric tonnes per hectare per year) and consumption district. Mbarara falls within the western medium–high farmlands agro‐ecological zone at an altitude ranging between 1400–1500 masl, with mean annual rainfall of up to 1223 mm and temperatures ranging from 12.5 ^◦^C to 30 ^◦^C.

### Tools, sampling and data collection

The study used a mixed method approach that included individual interviews,sex disaggregated focus group discussions (FGDs) and key informant interviews based on an adapted methodology in Forsythe *et al*, 2018 (step 2 manual). Mbarara district in western Uganda was selected as a representation of high banana production areas while Nakaseke District which is in central Uganda was selected as a representation of low production areas. In addition, these districts serve as site locations where the National Banana Research Program conducts evaluation of hybrid banana varieties. In each district, two sub‐counties were purposively selected, representing high (Ndeija and Kasangombe in Mbarara and Nakaseke respectively) and low banana production levels (Bubaare and Kito respectively). For each sub‐county, two parishes (four in total), and two villages within those parishes (8 in total), were randomly selected.

In each village; one key informant^2^ interview and two FGDs (one with men only and another with women only) were conducted. In addition, at least 10 individuals from each village were randomly selected for the individual interviews. In total, eight key informant interviews (two women, six men);^3^ 14 focus group discussions comprising 164 participants (Eight‐five women and Seventy nine men) and 123 individual interviews (Sixty‐four women and Fifty nine men) were conducted. All the participants produced, processed and/or consumed matooke.

For traders, lists of major banana urban markets were obtained from the district commercial officers, from which two markets per district were randomly selected. In each of the markets, a list of traders was obtained from market chairperson out of which a total of 40 traders (Fifteen women, Twenty‐five men) were sampled and interviewed.

Data collected included socioeconomic characteristics, *matooke* varieties grown and preferred by farmers and traders; and the varietal characteristics that drive preferences in the raw and steamed matooke product. The FGDs provided detailed information on norms and gender relations related to banana production, processing, consumption and preferences related to varietal characteristics at the different stages. An overview of the topics, tools and sampling is provided in Forsythe *et al*., 2018.

### Data analysis

Participants in the FGDs and individual interviews identified and ranked varieties and characteristics in order of importance (simple ranking). The rankings of varieties and characteristics were aggregated and weighted based on their rank to determine the overall ranks. To apply weights, the frequency (count) for the most important characteristic (1^st^ priority) was multiplied by *n* (rank), 2^nd^ priority by *n‐1* and the nth priority by 1. The weighted scores were then added to get summary scores for each characteristic which were used to rank the characteristics – the higher the score, the higher the rank (Forsythe *et al*., 2018). Chi‐square (*X* ^2^) tests were performed to test gender‐specific and regional differences. Excel and Stata v14 software were used for analyses.

## Results and discussion

### Demographic characteristics of farmers and traders from individual interviews

Most of the farmers were married (71%) and around 30.5% of women were widowed (Table 1). Almost all men (96%) were household heads. Men had slightly more average years of education compared to women (6.5 versus 5.6 years, respectively). The sample represented diverse ethnicities, which might influence values, customs and food preferences (Bechoff *et* *al*., 2020). Over 50% of farmers were from Baganda and Banyankole ethnic groups who are experts in matooke and often able to detect any quality deviations in introduced varieties. The term *matooke* is often used interchangeably with *food* (Hamilton *et al*., 2016), suggesting its importance in Ugandan diets.

**Table 1:** Demographic characteristics of respondents

	Characteristic		Women (%)	Men (%)	All (%)
Farmers	Marital status	N	64	59	123
		Divorced/separated	8.5	4.7	6.5
		Married/cohabiting	54.2	87.5	71.5
		Single/never married	6.8	6.3	6.5
		Widowed	30.5	1.6	15.5
	Ethnicity	Muganda	43.1	33.3	38.0
		Mukiga	‐	1.6	0.8
		Munyankole	44.8	61.9	53.7
		Munyarwanda	8.6	1.6	5.0
		Murundi	‐	1.6	0.8
		Musoga	1.7	‐	0.8
		Tanzanian	1.7	‐	0.8
	Main occupation	Farmer	96.6	96.9	96.8
		Non‐farm employment	3.4	3.1	3.3
	Relationship to household head	Daughter	1.7	‐	0.8
		Head	54.2	96.9	76.4
		Son	‐	1.6	0.8
		Spouse	44.1	1.6	22.0
	Education	Years (SD)	5.6 (3.7)	6.5 (3.7)	6.1(3.7)
	Age	Years (SD)	48.6 (14.3)	48.4 (15.2)	48.5 (14.7)
Traders	Marital status	N	6	11	17
		Divorced/separated	‐	9.1	11.8
		Married/cohabiting	83.3	81.8	82.4
		Single/never married	16.7	9.1	11.8
	Ethnicity	Muganda	16.7	27.2	23.5
		Munyankole	66.7	72.7	70.6
		Mukiga	16.7	‐	5.9
	Type of trader*	Retailers (stall)	83.3	9.1	35.2
		Wholesalers	33.3	72.7	58.8
		Motorcycle/bicycle	‐	27.3	17.7
	Level of education	None	‐	9.1	5.9
		Primary	66.7	63.6	64.7
		Secondary	33.3	27.3	29.4
	Age	Years (SD)	32.7 (11.5)	36.3 (6.6)	35.0 (8.5)
	No. of years in banana trading	Years (SD)	4.1 (2.2)	9.5 (7.7)	7.6 (6.7)

* A trader could fit into more than one category hence the percentages add to more than 100.

Most of the traders were married (82%) and had completed primary education (65%). Women traders had been in the trade for under half the average years that men had been (4.1 vs 9.5 years, respectively). All men traders used bicycles and motorcycles to trade (Table 1). As expected, the majority of interviewed women traders were retailers with stationery stalls that did not involve moving from point A to B. The wholesale node seems to be dominated by men (28 out of 40 wholesale traders were men. Nalunga *et al*. (2015) report similar results on traders for the above‐mentioned parameters.

### Preferred varieties and their characteristics

**Figure 1:**
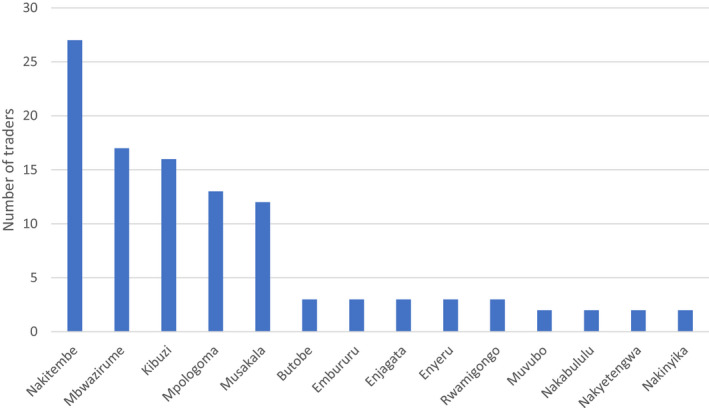
Varieties demanded by consumers, according to traders (*n *= 40).

Farmers were asked to rank the preferred banana varieties for making matooke. In Nakaseke, both landraces and hybrids were mentioned whereas in Mbarara, only landraces were reported. In both districts, the most preferred top five varieties are landraces (Table 2). Farmers prefer local varieties due to the superior quality characteristics (Nowakunda & Tushemereirwe, 2004; Akankwasa *et al*., 2016). Within the districts, there are gender differences in preferred varieties. More men (33%) in Mbarara mentioned *Mbwazirume* compared to only 4% of women (*P* = 0.007). Traders were each asked to rank the three main varieties that were demanded by their customers. Their answers correspond to the top varieties preferred by farmers for making steamed *matooke* (Fig. 1). The top six varieties ranked in order of frequency of mention by traders were: *Nakitembe*, *Mbwazirume*, *Kibuzi*, *Musakala*, *Mpologoma* and *Musakala*.

**Table 2:** Varieties preferred by farmers for making steamed mashed matooke (Citation Ranking %)

Variety	Mbarara			Nakaseke			ALL
	Citation Ranking (%)			Citation Ranking (%)			Frequency (%)
	Female	Male	Total	Female	Male	Total	
	*n *= 24	*n *= 39	*n *= 63	*n *= 25	*n *= 35	*n *= 60	*n *= 123
Nakitembe/Entaragaza (L)	45.8	33.3	38.1	54.3	32.0	45.0	41.5
Kibuzi (L)	37.5	53.9	47.6	8.6	8.0	8.3	28.5
Enyeru (L)	37.5	41.0	39.7	‐	‐	‐	20.3
Mbwazirume (L)	4.2^a^	33.3^a^	22.2	17.1	20.0	18.3	20.3
Musakala (L)	‐	2.6	1.6	34.3	16.0	26.7	13.8
Mpologoma (L)	‐	‐	‐	28.6	20.0	25.0	12.2
Enjagata (L)	12.5	10.3	11.1	‐	4.0	1.7	6.5
Muvubo (L)	12.5	2.6	6.4	5.7	8.0	6.7	6.5
Kisansa (L)	‐	‐	‐	5.7	20.0	11.7	5.7
Embururu (L)	4.2	2.6	3.2	11.4	4.0	8.3	5.7
Rwamigongo (L)	8.3	7.7	7.9	‐	‐	‐	4.1
Nakyetengu (L)	4.2	2.6	3.2	5.7	‐	3.3	3.3
Majaga (L)	‐	5.1	3.2	2.9	‐	1.7	2.4
Butobe (L)	‐	2.6	1.6	‐	4.0	1.7	1.6
Enzirabushera (L)	4.2	2.6	3.2	‐	‐	‐	1.6
Entukura (L)	‐	‐	‐	2.9	‐	1.7	0.8
FHIA (I)	‐	‐	‐	2.9	‐	1.7	0.8
Kabana (I)	‐	‐	‐	‐	4.0	1.7	0.8
Lusumba (L)	‐	‐	‐	2.9	‐	1.7	0.8
Nakawere (L)	‐	‐	‐	2.9	‐	1.7	0.8
Nakinyika (L)	‐	‐	‐	2.9	‐	1.7	0.8

Same letters in a row indicate significant association between men and women within a district using chi‐square tests at the 5% level.

* Local (L), Introduced hybrid (I). Variety names have synonyms and the same variety might be called a different name depending on the location. The main name and synonyms are: Nakitembe (syn. Entaragaza); Embururu (syn. Butende, Nakabululu); Musakala (syn. Mushakara, Nshakara); Rwamigongo (syn. Egongo); Enjagata (syn. Nandigobe, Njagata); Muvubo (syn., Mujuba); Enyeru (Nyeru)

The main characteristics that drive preferences by farmers are quality (soft, taste, yellow food) and agronomic such as big bunch, big fingers (Table 3). Quality characteristics are rated higher than agronomic characteristics by both men and women across districts. Other important characteristics considered mainly by women farmers include ease of peeling. Traders mention characteristics related to convenience during handling, transport and what customers demand.

**Table 3:** Characteristics varieties of preferred for making steamed matooke (Citation Ranking (%) of farmers and characteristics mentioned by traders)

	Female	Male	Mbarara	Nakaseke	Total
	*n *= 59	*n *= 64	*n *= 63	*n *= 60	*n *= 123
Gives soft food*	55.9	46.9	44.4	58.3	51.2
Gives tasty food*	37.3	29.7	27.0	40.0	33.3
Gives yellow food*	17.0	14.1	17.5	13.3	15.5
Produces big bunches*	17.0	10.9	9.5	18.3	13.8
Produces big fingers*	15.3	12.5	14.3	13.3	13.8
Has good smell*	6.8	12.5	9.5	10.0	9.8
Easy to peel	10.2	4.7	4.8	10.0	7.3
Produces long fingers*	8.5	6.3	6.4	8.3	7.3
Easy to cook/ fast cooking	5.1	6.3	4.8	6.7	5.7
Gives firm steamed food	5.1	4.7	9.5^a^	0.0^a^	4.9
Produces starchy food	3.4	6.3	4.8	5.0	4.9
Produce round fingers	5.1	3.1	4.8	3.3	4.1
Long shelf life*	1.7	0.0	0.0	1.7	0.8
Shiny fingers*	1.7	0.0	0.0	1.7	0.8

Same letters in row indicate significant association between district or sex using chi‐square tests at the 5% level.

* Traits also mentioned by traders.

**Figure 2:**
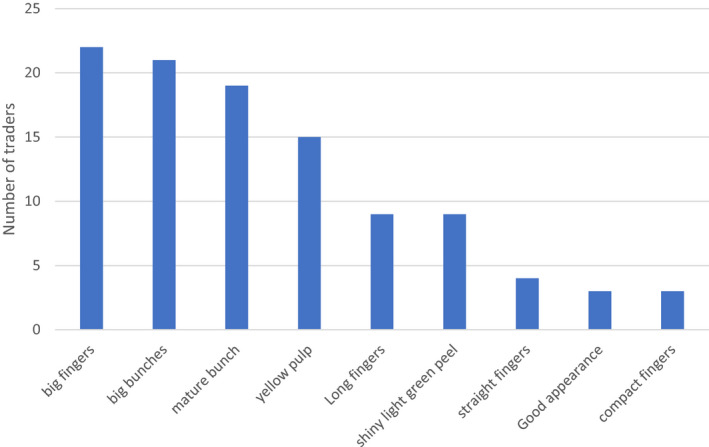
Traders’ perceptions of characteristics preferred by consumers (*n *= 40).

For traders, the top five characteristics they perceive to be important for their customers are big fingers, big bunches, maturity, shiny light green peel colour, good appearance (fresh, appealing, good finger formation) and compact bunches/fingers (Fig. 2). Other mentioned characteristics which cater for the diverse customers served include long fingers, medium bunches, medium sized fingers, straight fingers and varieties that are soft when cooked. In addition, traders also mentioned variety type (local is preferred), price (buying vs selling margin), quality of bunch and fingers (good quality are big and attractive), size of bunch/fingers, maturity level (not very mature preferred to avoid over‐ripening), appearance (fresh, smooth, no bruises, disease free), finger shape, bunch shape (cylindrical), transaction costs (e.g. fuel costs) and location.

### Preferred and not‐preferred characteristics of the raw material (bunch and fingers)

Both women and men agree on the importance of most of the characteristics and therefore rank them the same way (Tables 4 and 5). Big bunches, big fingers with a light green colour and a yellowish pulp colour are preferred by both men and women farmers (Table 4). At the sites, both men and women ranked small finger size as their number one attribute that is not desired in matooke. This could be because finger size is a key market requirement and men are the ones involved in selling; but also, small fingers are difficult to peel, an activity mainly done by women. Women ranked pulp colour higher compared to men. This could be attributed to the fact that women are the ones involved in preparation of food for the households and take keener interest in the quality of food compared to men. However, women and men agree on the importance of most of the characteristics and therefore rank them in the same way (Table 5).

**Table 4:** Scores for characteristics of raw material (bunch and fingers) that produce good quality steamed matooke determined at harvest (Citation Ranking (%) of farmers)

	Female	Male	Mbarara	Nakaseke	Total
	*n *= 59	*n *= 64	*n *= 63	*n *= 60	*n *= 123
Big fingers	64.4	51.6	60.3	55.0	57.7
Big bunch	33.9	35.9	34.9	35.0	35.0
Yellowish/ creamish pulp colour	28.8	37.5	30.2	36.7	33.3
Shiny light green finger colour	42.4^a^	25.0^a^	34.9	31.7	33.3
Disease free/spotless fingers	6.8	17.2	9.5	15.0	12.2
Long fingers	10.2	10.9	6.4	15.0	10.6
Low sap content	5.1	9.4	6.4	8.3	7.3
Compact fingers	6.8	4.7	7.9	3.3	5.7
Thin peel	5.1	3.1	1.6	6.7	4.1
Big peduncle	3.4	3.1	6.4^a^	0.0^a^	3.3
High sap content	6.8^a^	0.0^a^	4.8	1.7	3.3
Soft when touched	3.4	3.1	0.0^a^	6.7^a^	3.3
Soft peel	3.4	1.6	1.6	3.3	2.4
Fingers are not cracked	0.0	1.6	0.0	1.7	0.8

Same letters in row indicate significant association between district or sex using chi‐square tests at the 5% level.

**Table 5:** Rankings and summary scores of characteristics that are not preferred generally in matooke (Citation Ranking %)

	Female	Male	Mbarara	Nakaseke	Total
	*n *= 59	*n *= 64	*n *= 63	*n *= 60	*n *= 123
Small short fruits	50.9	48.4	50.8	48.3	**49.6**
Spotted/diseased	30.5	31.3	19.1^a^	43.3^a^	**30.9**
White pulp colour	22.0	20.3	27.0	15.0	**21.1**
Hard/ brittle fruits	18.6	21.9	20.6	20.0	**20.3**
Hard to peel	11.9	9.4	12.7	8.3	**10.6**
Small bunch	10.2	7.8	6.4	11.7	8.9
Cracked fruits	5.1	9.4	4.8	10.0	7.3
High sap content	6.8	6.3	3.2	10.0	6.5
Plant looks weak, unhealthy	6.8^a^	0.0^a^	1.6	5.0	3.3
No/little sap	6.8	3.1	7.9	1.7	4.9
Poor appearance	3.4	1.6	1.6	3.3	2.4
Separated fingers	0.0	4.7	4.8	0.0	2.4
Yellow pseudostem colour	5.1	0.0	4.8	0.0	2.4
Small pseudostem	1.7	1.6	3.2	0.0	1.6
Takes long to cook	1.7	1.6	3.2	0.0	1.6
Curved fruits	1.7	0.0	1.6	0.0	0.8

Bold values indicate the top most five characteristics that are not preferred. Values with letters indicate differences across gender and geographical regions.

The top five undesired attributes are small and short fingers, immature fruits, spotted/diseased fruits, hard/ brittle fingers, white/creamish pulp colour and hard to peel fruit (Table 5). Similar findings are reported in Akankwasa *et al*. (2016) and Nalunga *et al*. (2015). Small and short fingers were mentioned by all the respondents at all the sites as undesired characteristics. Finger size is a key market requirement and small fingers are reported to be difficult to peel. More men (80%) in Mbarara compared to Nakaseke (71%) mentioned big finger size as an important characteristic. This could be because in Mbarara, banana production is more commercialised than Nakaseke and men are more involved in selling.

Women ranked pulp colour higher compared to men. A yellowish pulp colour is associated with good food colour. Food preparation is mostly women’s responsibility (Rietveld & Farnworth, 2018; Marimo *et al*., 2019; Weltzien *et al*., 2020), hence the need to ensure that new varieties are easy to peel, as well as further research on other gendered characteristics. Women particularly pay more attention to postharvest processing and food quality characteristics in a range of crops hence the need to consider gender‐specific characteristics to improve varietal acceptance and end‐user benefits (Weltzien *et al*., 2020). Farmers in western Uganda are more sensitive to pulp colour compared to those in central. This could be because there is less production in central Uganda and consumers tend to have fewer options and therefore are less selective.

Results of the focus group discussions (Table 6) in both districts ranked maturity of the cooking bananas as the most important characteristic. Women groups ranked big fingers and creamy pulp colour as the second most important characteristics, as was observed with individual interviews. Similarly, men groups ranked big bunch and disease free as their preferred characteristics. Generally, results of focus group discussions are in agreement with the individual interviews.

**Table 6:** Ranked scores of characteristics of raw material (bunch and fingers) that produce good quality steamed matooke determined at harvest farmers from focus group discussions

Attribute	Female		Male		Nakaseke		Mbarara	
	Score	Rank	Score	Rank	Score	Rank	Score	Rank
Shiny green colour	9	2^nd^	7	2^nd^	7	2^nd^	8	2^nd^
Variety specific	2	4^th^	2	5^th^	4	3^rd^	1	6^th^
Disease free	0	6^th^	3	4^th^	1	6^th^	2	5^th^
Mature enough	21	1^st^	16	1^st^	14	1^st^	23	1^st^
Breaks at harvesting	0	6^th^	5	3^rd^	3	4^th^	4	3^rd^
Big bunch	0	6^th^	5	3^rd^	3	4^th^	0	7^th^
Much sap	2	4^th^	0	6^th^	0	7^th^	2	5^th^
Creamy pulp colour	3	3^rd^	2	5^th^	2	5^th^	3	4^th^
Big fingers	3	3^rd^	2	5^th^	4	3^rd^	1	6^th^
Tips fall off	1	5^th^	0	6^th^	1	6^th^	0	7^th^

### Characteristics that are important at the different stages of cooking matooke

Table 7 summarises characteristics mentioned by farmers from harvest of bunches up to the preparation of steamed mashed matooke. At the peeling stage, important characteristics include easy to peel, straight fruits, soft peel, soft pulp, yellowish/creamish pulp colour and low sap content. During washing, matooke processors prefer varieties with a low amount of sap. However, for steaming and simmering, no specific characteristics are important.

**Table 7:** Summary of key characteristics at each stage of processing steamed mashed matooke

Steps in matooke preparation	Key characteristics
1. Harvesting, cut a fully‐grown banana bunch(es)	Mature big bunch, compact bunch/fingers
2. De‐hand ‐remove hands from bunch and remove fingers from clusters	Well filled big fingers, yellowish/creamish pulp colour, shiny light green peel colour, disease free/spotless, long fingers
3. Peeling	Easy to peel, yellowish pulp
4. Washing	Sap content (can be high or low) depending on consumer perceptions
5. Prepare saucepan – put strips of banana fibres and stalks as a foundation at the bottom of a cooking pan to avoid the boiling water touching the bundle of matooke being steamed.	None
6. Prepare leaves – carefully slice off the midribs	None. Characteristics at this stage are related to the leaves and not the raw material. Leaves that can fold easily e.g. from *Sukali Ndizi* and those from *Kayinja* which is perceived to influence aroma are preferred
7. Tying up the peeled and washed banana fingers in a bundle of banana leaves	None required
8. Place tied bundle into a cooking pot on top of the fibres and/or stalks with enough water to steam the leaves.	None required
9. Steaming for about 1hr? – depends on the type of firewood	None required
10. After steaming, smash cooked bananas by pressing with the palms of one’s hands to make matooke.	None. Processors indicate there are no particular characteristics and no differences among varieties during the pressing step
11. Let the matooke simmer for a little bit	None required
12. Serving matooke	Preferred characteristics of high quality steamed mashed matooke by both men and women in the two districts include soft texture, good smell, yellow colour, good matooke taste and compact in that order

### Preferred and not‐preferred characteristics of matooke ‐ the final cooked product

Soft texture, good aroma, yellow colour and good taste are the four top preferred characteristics of steamed matooke (Table 8a). Similar results were obtained from focus group discussions (Table 9). Ssemwanga (1996); Nowakunda *et al*. (2000); Nowakunda & Tushemereirwe (2004); Akankwasa *et al*. (2013) reported that hard texture, astringent taste, poor aroma and lack of golden yellow colour often led to rejection of matooke hybrids by end‐users. Women seem to be more sensitive to appearance than men while the men are more sensitive to taste than women. This could be explained by the fact that women are the ones who are more responsible for household food and are therefore more sensitive to quality whereas the men are more interested in market preferred characteristics. These results also show that there are gender differences in the preference for banana characteristics. According to Weltzien *et al*. (2020), trait preferences differ because women and men have contrasting roles and responsibilities for various crop production or postharvest activities. Bellon and Reeves (2002) suggests that there are gender differences in the demand for particular characteristics and failure to recognise these differences would lead to biased interventions. The less preferred characteristics include hard texture, too soft or watery matooke, pale yellow colour and flat taste (Table 8b). Consumers in Nakaseke ranked watery/too soft texture as number 4 while it was undesired attribute number 2 in Mbarara. Similar to the explanation for preferences for the raw product, this regional difference could be explained by limited access to different product options for Nakaseke consumers.

**Table 8:** Characteristics of (a) good quality (b) poor quality steamed mashed matooke (Citation Ranking %)

	Female	Male	Mbarara	Nakaseke	Total
	*n *= 59	*n *= 64	*n *= 63	*n *= 60	*n *= 123
(a)					
Soft texture	88.1	82.8	76.2^a^	95.0^a^	85.4
Good smell	76.3	71.9	79.4	68.3	74.0
Yellow colour	59.3	51.6	46.0^a^	65.0^a^	55.3
Good matooke taste	44.1	50.0	49.2	45.0	47.2
Holds together when mashed (compact)	25.4	21.9	30.2	16.7	23.6
Elastic/starchy	20.3	15.6	11.1^a^	25.0^a^	17.9
Uniform/homogenous texture	10.2	6.3	12.7	3.3	8.1
Smooth mouthfeel	5.1	9.4	11.1	3.3	7.3
Does not separate/break when served	5.1	3.1	6.4	1.7	4.1
Not sticky	3.4	4.7	6.4	1.7	4.1
Homogenous colour	3.4	3.1	0.0^a^	6.7^a^	3.3
Does not harden quickly	1.7	3.1	1.6	3.3	2.4
Satisfying	3.4	0.0	1.6	1.7	1.6
(b)					
Hard	59.3	70.3	60.3	70.0	65.0
Watery	37.3	32.8	39.7	30.0	35.0
White colour	25.4	25.0	34.9^a^	15.0^a^	25.2
Separates easily/ not compact	22.0	26.6	25.4	23.3	24.4
Poor/flat taste	27.1	15.6	12.7^a^	30.0^a^	21.1
No steamed banana smell	13.6	12.5	7.9	18.3	13.0
Blackish colour	11.9	12.5	9.5	15.0	12.2
Non‐homogenous	6.8	1.6	0.0^a^	8.3^a^	4.1
Not yellow in colour	6.8	1.6	0.0	8.3	4.1
Non‐homogenous texture	5.1	1.6	4.8	1.7	3.3
Brownish colour	1.7	1.6	3.2	0.0	1.6
Cools fast after serving	1.7	1.6	0.0	3.3	1.6
With thread like materials	1.7	0.0	0.0	1.7	0.8

Same letters in a row indicate significant association between district or sex using chi‐square tests at the 5% level.

**Table 9:** Ranked scores of characteristics of good quality steamed mashed matooke from focus group discussions

Attribute	Female		Male		Nakaseke		Mbarara	
	Score	Rank	Score	Rank	Score	Rank	Score	Rank
Soft texture	15	1^st^	11	1^st^	11	1^st^	15	1^st^
Good local matooke taste	0	5^th^	2	5^th^	2	5^th^	0	5^th^
Aroma of steamed matooke	8	3^rd^	7	3^rd^	7	3^rd^	8	3^rd^
Elastic	0	5^th^	2	5^th^	2	5^th^	0	5^th^
smooth mouth feel	0	5^th^	2	5^th^	2	5^th^	0	5^th^
Yellow colour	13	2^nd^	9	2^nd^	9	2^nd^	13	2^nd^
Compact/firm	3	4^th^	7	3^rd^	7	3^rd^	3	4^th^

## Conclusion

This study showed that famers and traders in the western Uganda food chain for matooke give priority to characteristics that influence market acceptance. In the central region, consumers attach less importance to food colour compared to the western region. Generally, traders look for characteristics that buyers ask for when purchasing the banana bunches.

Women and men mention the same characteristics with minimal differences in the proportions reporting certain characteristics and in the assigned rankings. More women mention characteristics related to the preparation process for example ease of peeling, thin peel and soft peel as they are mostly responsible for food preparation. Colour of the peel‐shiny light green fruits, and sap content are also important attributes for women.

Farmers and traders of matooke prefer local landraces because of their superior quality attributes compared with hybrids. However, these landraces are susceptible to pests and diseases and their productivity is low.

Soft texture, good aroma, yellow colour, good *matooke taste* and matooke that holds together when mashed are generally important characteristics for matooke end‐users. Indeed, varieties that lack these characteristics are often rejected. Characteristics that are not liked include; hard texture, too soft or watery matooke, pale yellow colour and flat taste. Varieties with such characteristics are often rejected.

Multidisciplinary efforts are encouraged in development of product profiles that include key user preferences, and their use during selection of hybrids. This will ensure user‐responsive breeding that holistically caters to all target food chain actors and segments.

It is imperative that the identified characteristics be associated with physical–chemical characteristics and translated into high‐throughput phenotyping tools such as NIRS^4^, for efficient and effective selection of user‐acceptable hybrids.

## Author contribution

**Kephas Nowakunda:** Conceptualization (equal); Data curation (equal); Formal analysis (equal); Funding acquisition (equal); Investigation (equal); Methodology (equal); Project administration (equal); Writing‐review & editing (equal). **Kenneth Akankwasa:** Conceptualization (equal); Data curation (equal); Formal analysis (equal); Investigation (equal); Methodology (equal); Writing‐original draft (equal); Writing‐review & editing (equal). **Pricilla Marimo:** Conceptualization (equal); Data curation (equal); Formal analysis (equal); Writing‐original draft (equal). **Robooni Tumuhimbise:** Conceptualization (equal); Writing‐original draft (equal); Writing‐review & editing (equal). **Moureen Asasisa:** Data curation (equal); Investigation (equal); Writing‐original draft (equal). **Elizabeth Khakasa:** Data curation (equal); Investigation (equal); Writing‐original draft (equal). **Innocent Mpiriirwe:** Data curation (equal); Formal analysis (equal). **Uli Kleih:** Data curation (equal); Methodology (equal); Writing‐original draft (equal). **Lora Forsythe:** Conceptualization (equal); Data curation (equal); Methodology (equal). **Genevieve Fliedel:** Data curation (equal); Methodology (equal); Writing‐original draft (equal). **Dominique Dufour:** Conceptualization (equal); Methodology (equal); Writing‐review & editing (equal).

## Conflict of interest

The authors declare no conflict of interest in this work.

## Ethical approval

This study was approved by the National Research Ethics Committee accredited by the Uganda National Council for Science and Technology. Research teams obtained ethical approval prior to the fieldwork. Informed consent was sought from all participants before conducting any activities and it was emphasised that they could stop the interview at any point without any penalty. The study respected the rules of voluntary participation and anonymity.

### Peer Review

The peer review history for this article is available at https://publons.com/publon/10.1111/ijfs.14813.

## Data Availability

Data is available on request from the authors.

## Notes

^1^ ‘Matooke’ can synonymously refer to EAHCB or the steamed‐mashed food made from the bananas. In this article the word will be used interchangeably.

^2^ Key informants were purposively selected individuals holding key leadership positions and/or had expertise/ knowledge of the community– most were men. Our sample included community leaders, a key farmer, an extension officer, a youth group leader, and a farmer group leader.

^3^ In one of the villages separate men only and women only FGDs could not be conducted due to logistical reasons hence a joint discussion was done.

^4^ Near‐infrared spectroscopy (NIRS).
